# Comparative study of the gut microbiota in three captive *Rhinopithecus* species

**DOI:** 10.1186/s12864-023-09440-z

**Published:** 2023-07-14

**Authors:** Li Xi, Xiaohui Wen, Ting Jia, Jincheng Han, Xinxi Qin, Yanzhen Zhang, Zihan Wang

**Affiliations:** 1 College of Biology and Food, Shangqiu Normal University, Shangqiu, China; 2 Henan Engineering Research Center of Development and Application of Green Feed Additives, Shangqiu Normal University, Shangqiu, China; 3 Institute of Animal Health, Guangdong Academy of Agricultural Sciences, Guangzhou, China; 4 Beijing Key Laboratory of Captive Wildlife Technologies, Beijing Zoo, Beijing, China; 5 College of Veterinary Medicine, Northwest A&F University, Yangling, China

**Keywords:** Snub-nosed monkey, Gut microbiota, Captivity, Species, Conservation

## Abstract

**Background:**

Snub-nosed monkeys are highly endangered primates and their population continues to decline with the habitat fragmentation. Artificial feeding and breeding is an important auxiliary conservation strategy. Studies have shown that changes and imbalances in the gut microbiota often cause gastrointestinal problems in captive snub-nosed monkeys. Here, we compare the gut microbiota composition, diversity, and predicted metabolic function of three endangered species of snub-nosed monkeys (*Rhinopithecus bieti*, *R. brelichi*, and *R. roxellana*) under the same captive conditions to further our understanding of the microbiota of these endangered primates and inform captive conservation strategies. 16 S rRNA gene sequencing was performed on fecal samples from 15 individuals (*R. bieti* N = 5, *R. brelichi* N = 5, *R. roxellana* N = 5).

**Results:**

The results showed that the three *Rhinopithecus* species shared 24.70% of their amplicon sequence variants (ASVs), indicating that the composition of the gut microbiota varied among the three *Rhinopithecus* species. The phyla Firmicutes and Bacteroidetes represented 69.74% and 18.45% of the core microbiota. In particular, analysis of microbiota diversity and predicted metabolic function revealed a profound impact of host species on the gut microbiota. At the genus level, significant enrichment of cellulolytic genera including *Rikenellaceae RC9 gut group*, *Ruminococcus*, *Christensenellaceae R7 group*, *UCG 004* from Erysipelatoclostridiaceae, and *UCG 002* and *UCG 005* from Oscillospiraceae, and carbohydrate metabolism including propionate and butyrate metabolic pathways in the gut of *R. bieti* indicated that *R. bieti* potentially has a stronger ability to use plant fibers as energy substances. *Bacteroides*, *unclassified Muribaculaceae*, *Treponema*, and *unclassified Eubacterium coprostanoligenes group* were significantly enriched in *R. brelichi*. *Prevotella 9*, *unclassified Lachnospiraceae*, and *unclassified UCG 010* from Oscillospirales UCG 010 were significantly enriched in *R. roxellana*. Among the predicted secondary metabolic pathways, the glycan biosynthesis and metabolism had significantly higher relative abundance in the gut of *R. brelichi* and *R. roxellana* than in the gut of *R. bieti*. The above results suggest that different *Rhinopithecus* species may have different strategies for carbohydrate metabolism. The Principal coordinate analysis (PCoA) and Unweighted pair-group method with arithmetic mean (UPGMA) clustering tree revealed fewer differences between the gut microbiota of *R. brelichi* and *R. roxellana*. Correspondingly, no differences were detected in the relative abundances of functional genes between the two *Rhinopithecus* species.

**Conclusion:**

Taken together, the study highlights that host species have an effect on the composition and function of the gut microbiota of snub-nosed monkeys. Therefore, the host species should be considered when developing nutritional strategies and investigating the effects of niche on the gut microbiota of snub-nosed monkeys.

**Supplementary Information:**

The online version contains supplementary material available at 10.1186/s12864-023-09440-z.

## Background

The gut microbiota forms a complex ecosystem through bacterial interactions, which widely affects the physiological structure and function of the host gut. Gut microbiota can prevent or inhibit the invasion of pathogenic bacteria by producing bacteriocins, organic acids, hydrogen peroxide and other substances, thus producing non-specific immune effect. In addition, the gut microbiota can be used as an antigen to stimulate and promote the development and maturation of the host immune system, enabling the animal body to obtain resistance to many pathogenic bacteria and their toxins, thus exerting a specific immune effect [1, 2]. Gut microbes are important players in host metabolism, providing substrate, enzymes, and energy [3–5]. At the same time, colonization of gut microorganisms is largely restricted and regulated by the host’s physiological structure and immune system, and the composition of the gut microbiota will change with the host’s physiological state, food, and habitat [6]. Mutualism between the host and its gut microbiota is believed to be created by their mutual adaptation and selection during a long period of co-evolution [7, 8].

Golden monkeys, also known as snub-nosed monkeys, are endemic to some montane forests in China and Vietnam. Snub-nosed monkeys include five endangered species according to the IUCN (International Union for Conservation of Nature): *R. bieti*, *R. brelichi*, *R. roxellana*, *R. strykeri*, and *R. avunculu*. Studies have confirmed that the northern species (*R. brelichi* and *R. roxellana*) and the Himalayan species (*R. bieti* and *R. strykeri*) diverged about 1.6 million years ago [9]. The *R. bieti*, *R. brelichi*, and *R. roxellana* are endemic species in China. *R. bieti* is the largest monkey in the genus *Rhinopithecus*, living in virgin alpine forests at an altitude of 2,500-5,000 m in southeastern Tibet and northwestern Yunnan [10]. *R. brelichi* live in forests at an altitude of 500–800 m on Fanjing Mountain, Guizhou Province [11]. *R. roxellana* is distributed in forests at an altitude of 1,500-3,300 m in Sichuan, Gansu, Shaanxi and Hubei provinces of China [12]. In terms of feeding habits, *R. bieti* and *R. brelichi* mainly eat plant food (leaf, shoot, bud, fruit, bark, and lichen) [13, 14]. *R. roxellana* has a relatively diverse diet. In addition to plant food, they also occasionally prey on birds, eggs, small animals, or insects [15]. Snub-nosed monkeys have a highly specialized stomach with internal septation, similar to a ruminant stomach. The abundant cellulolytic bacteria in the stomach give snub-nosed monkeys a powerful ability to digest the leaves, stems, and bark of plants [9]. In particular, the composition and abundance of the foregut and hindgut microbiota in snub-nosed monkeys varies significantly, and the expression of functional genes related to fiber digestion is higher in the foregut. However, both the foregut and the hindgut were dominated by bacterial communities capable of producing complex carbohydrate-degrading enzymes [16]. Such physiological characteristics give them a strong ability to digest plant fibers and to tolerate seasons or environments lacking fruit for a long time.

Currently, researchers are focusing more on the characteristics of the gut microbiota of snub-nosed monkeys in different habitats particularly in captive vs. wild animals. Hale et al. (2019) found that the richness of the gut microbiota was higher in wild *R. brelichi* than in captive *R. brelichi*. Lachnospiraceae and Ruminococcaceae, which can digest complex plant fibers and produce butyrate, were significantly enriched in the gut of wild *R. brelichi*. In contrast, the captive *R. brelichi* gut was enriched with more genera of *Prevotella* and *Bacteroides* capable of degrading simple sugars and carbohydrates [17]. Comparison of wild and captive *R. roxellana* showed that the richness and evenness of the gut microbiota were significantly higher in captive *R. roxellana*. The ratio of Prevotella/Bacteroides was significantly increased in the captive *R. roxellana*, suggesting an increased ability to digest simple sugars. The significantly decreased abundance of Firmicutes and the enrichment of genes involved in the pentose phosphate pathway and glutamate biosynthesis all indicated a weakening of fiber degradation ability in *R. roxellana*. Flexible adjustment of gut microbiota could allow *R. roxellana* to better adapt to dietary changes in captivity [18].

Furthermore, research also demonstrates that a similar diet can lead to convergence in the composition or function of the microbiota in primates. Under the same captive conditions, the phylogenetically distant *Colobus guereza* (African colobine) shared a similar gut microbiota composition with *Rhinopithecus* and *Trachypithecus* (Asian colobine), suggesting that similar diets can lead to convergence of the gut microbiota [19]. Li et al. (2023) and Xia et al. (2022) found that the function of the gut microbiota of wild *R. roxellana* and *R. bieti* converged after artificial food supply (including peanuts, apples, carrots, etc.). The gut microbiota composition of *R. roxellana* was quite different before and after food supply. Firmicutes abundance decreased significantly after food provision, along with an increase in Bacteroidetes. At the genus level, 1,965 ASVs were unique to the unfed group, while only 137 and 178 ASVs were unique to the food-provisioned groups. Similarly, there were obvious differences in the composition of the gut microbiota of *R. bieti* before and after food provision. When comparing the differences in the gut microbiota between *R. roxellana* and *R. bieti*, it was found that the host species had more profound effects on the gut microbiota than the diet. However, the PCoA results obtained by the Bray-curtis distance algorithm based on the abundance of KEGG pathway enzymes showed that the gut microbiota function of *R. roxellana* and *R. bieti* was relatively distant before food provision, but clustered after food provision [20, 21]. Combined with the above results, it can be seen that artificial feeding of similar food can lead to convergence of gut microbiota function.

With habitat fragmentation and tourism development, the number of endangered snub-nosed monkeys continues to decline, so the task of protecting these monkeys is urgent. In addition to the establishment of a natural reserve, artificial feeding is also one of the most important means of conserving snub-nosed monkeys. Captivity changes the diet structure, living environment, and habits of the monkeys, and then affects the health and gut microbiota of the monkeys. In recent years, there have been many reports on comparison of the gut microbiota of wild and captive snub-nosed monkeys. However, the characteristics of the gut microbiota of the endemic and endangered snub-nosed monkeys in China under the same captive conditions have not been reported. Here, we address these gaps in knowledge and use 16 S rRNA gene sequencing to compare the gut microbiota of three species of captive Golden monkeys (*R. bieti* N = 5; *R. brelichi* N = 5; *R. roxellana* N = 5) housed in the Beijing Zoo. We first identify the bacterial ASVs that are shared across *Rhinopithecus* species and then determine whether differences are observed in their microbiota diversity, composition, and predicted metabolic function. The characteristics of the gut microbiota obtained from the study can provide reference for disease surveillance and dietary adjustment during captive conservation of snub-nosed monkeys.

## Results

### Sequencing information

**Fig. 1:**
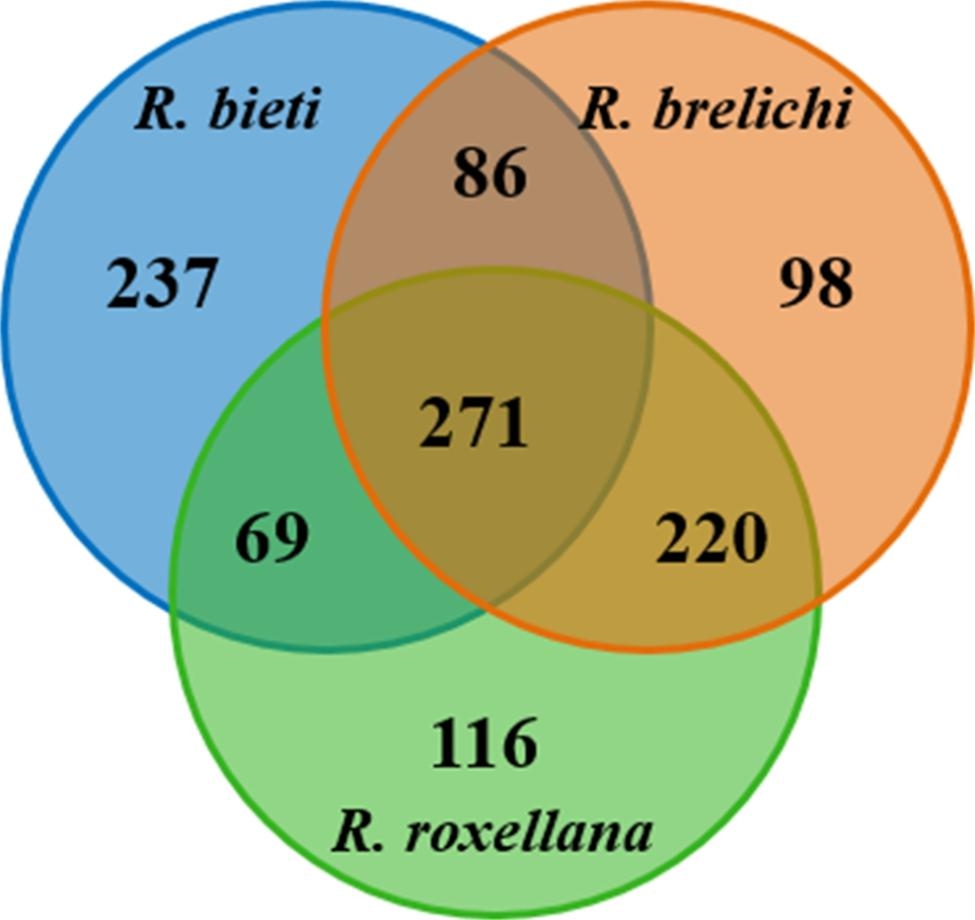
Shared and unique ASVs among the three *Rhinopithecus* species were visualized by Venn diagram

A total of 1,204,090 paired-end reads were generated from 15 samples from 3 *Rhinopithecus* species. 1,200,702 clean reads were obtained after quality control and assembly (Additional file 1). The three *Rhinopithecus* species clustered 1,097 ASVs. The Venn diagram showed that 271 ASVs present in all 15 samples of the three *Rhinopithecus* species, which mainly in Firmicutes (189 ASVs) and Bacteroidetes (50 ASVs). The core families were mainly composed of Oscillospiraceae, Lachnospiraceae, Christensenellaceae, unclassified Bacteroidales, Ruminococcaceae, uncultured rumen bacterium, UCG 010, Muribaculaceae, Rikenellaceae, Prevotellaceae, and Bacteroidaceae. The number of unique ASVs was 237 for *R. bieti*, 98 for *R. brelichi*, and 116 for *R. roxellana*, respectively (Fig. 1). With increasing sequencing volume (Additional file 2), the Rarefaction curve flattened and the library coverage of all samples was above 99.90%, indicating that the sequencing volume is sufficient to cover all samples.

### Analysis of the gut microbiota diversity

**Fig. 2:**
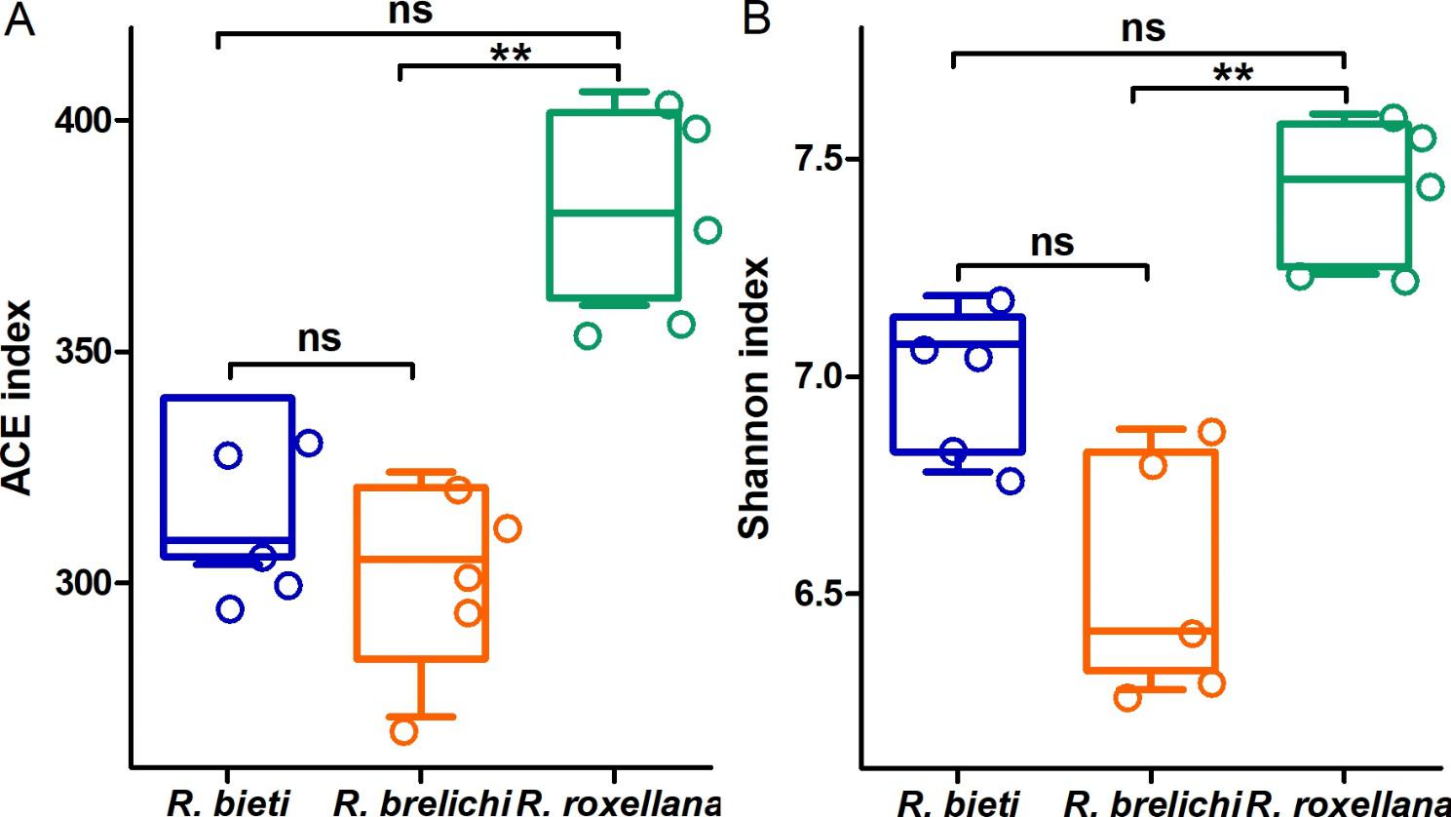
Differences in Alpha diversity of gut microbiota among the three *Rhinopithecus* species. **A** Pairwise comparisons of the ACE index among *Rhinopithecus* species. **B** Pairwise comparisons of the Shannon index among *Rhinopithecus* species. Kruskal Wallis rank-sum test, and *P*-values were corrected using the Benjamini-Hochberg method. ns: *P* > 0.05, no significance; * *P* < 0.05; ** *P* < 0.01

**Fig. 3:**
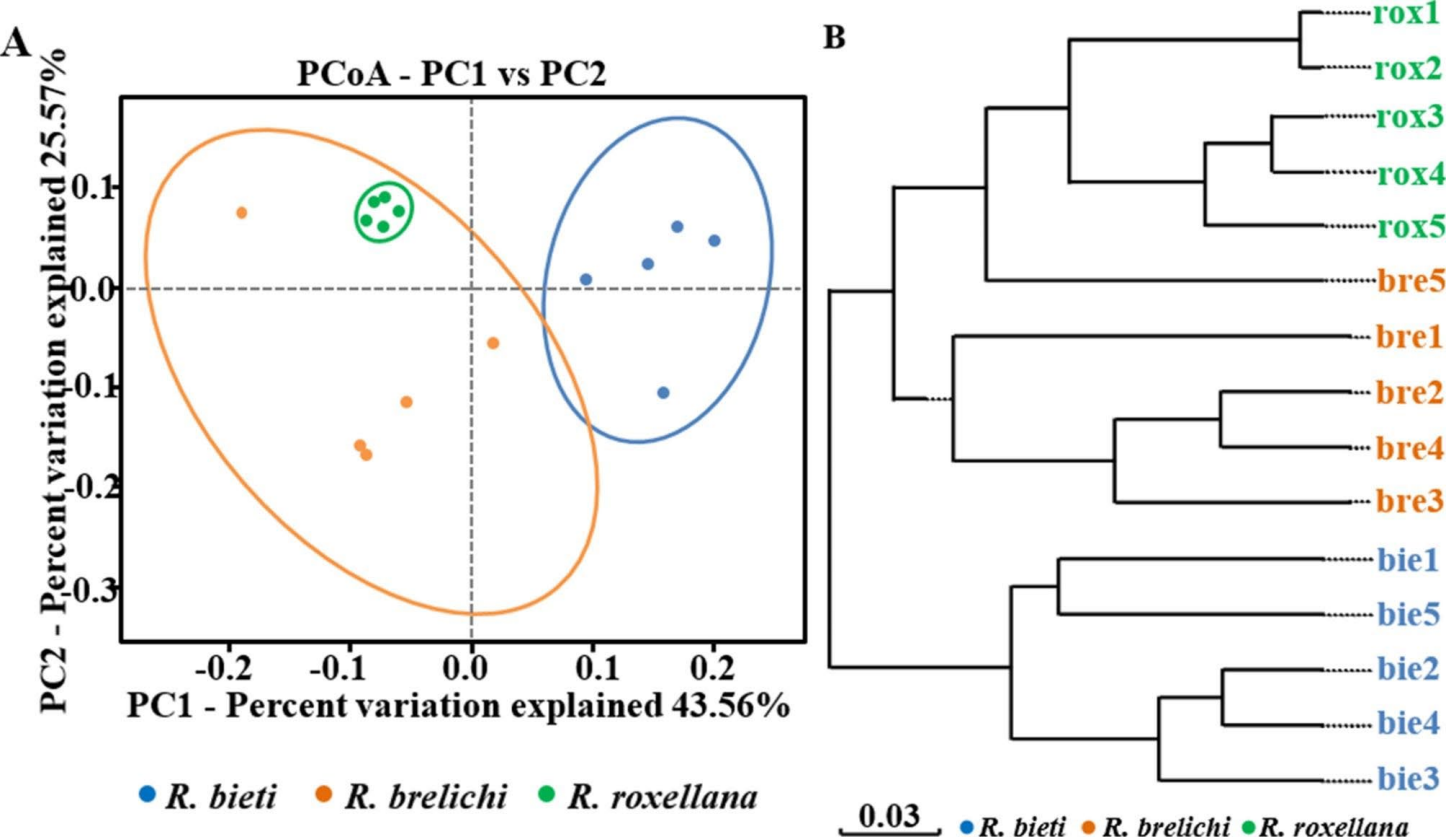
Beta diversity of the gut microbiota of the three *Rhinopithecus* species. **A** PCoA plot. **B** UPGMA clustering tree. Weighted UniFrac algorithm

The Alpha diversity index reflects the richness and evenness of the microbiota composition of the samples. As shown in Fig. 2, the ACE and Shannon index of the gut microbiota of *R. roxellana* were significantly higher than *R. brelichi* (ACE: 381.25 vs. 302.63, *P* < 0.01; Shannon: 7.42 vs. 6.54, *P* < 0.01). There were no significant differences in the ACE index and Shannon index between *R. bieti* and *R. brelichi* (ACE: 320.07 vs. 302.63, *P* > 0.05; Shannon: 7.00 vs. 6.54, *P* > 0.05) and between *R. bieti* and *R. roxellana* (ACE: 320.07 vs. 381.25, *P* > 0.05; Shannon: 7.00 vs. 7.42, *P* > 0.05). The PCoA plot drawn using a weighted UniFrac distance matrix demonstrated the microbiota distance between the samples. The results showed that the three *Rhinopithecus* species had significantly different microbiota compositions (Fig. 3A), and this difference had been proved by permutational multivariate analysis of variance (PERMANOVA) analysis (*R. bieti* vs. *R*. *brelichi*: *R*^*2*^ = 0.478, *P* = 0.001; *R. bieti* vs. *R. roxellana*: *R*^*2*^ = 0.590, *P* = 0.001; *R. brelichi* vs. *R. roxellana*: *R*^*2*^ = 0.324, *P* = 0.016) and analysis of similarities (ANOSIM) (*R. bieti* vs.  *R*. *brelichi*: *R* = 0.792, *P* = 0.007; *R. bieti* vs. *R. roxellana*: *R* = 1.000, *P* = 0.007; *R. brelichi* vs. *R. roxellana*: *R* = 0.492, *P* = 0.030). On the basis of the weighted UniFrac distance matrix, the samples were hierarchically clustered by UPGMA to determine the phylogenetic relationships between microbes among the samples. The results revealed a more similar species composition between the gut microbiota of *R. brelichi* and *R. roxellana* (Fig. 3B).

### Characteristics of the gut microbiota composition

**Fig. 4:**
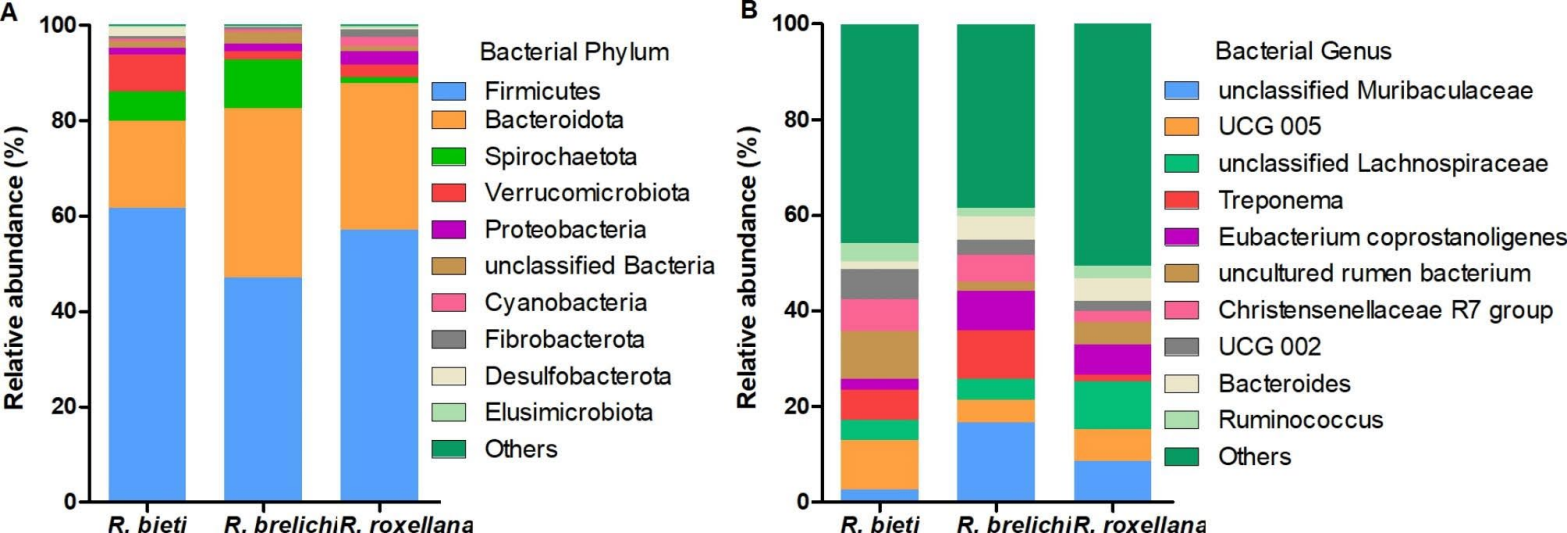
Species distribution of the gut microbiota of the three *Rhinopithecus* species. **A** The distribution histogram of the top ten phyla in the three species. **B** The distribution histogram of the top ten genera in the three species. The relative abundances of phyla and genera in the figure refers to the average value

**Fig. 5:**
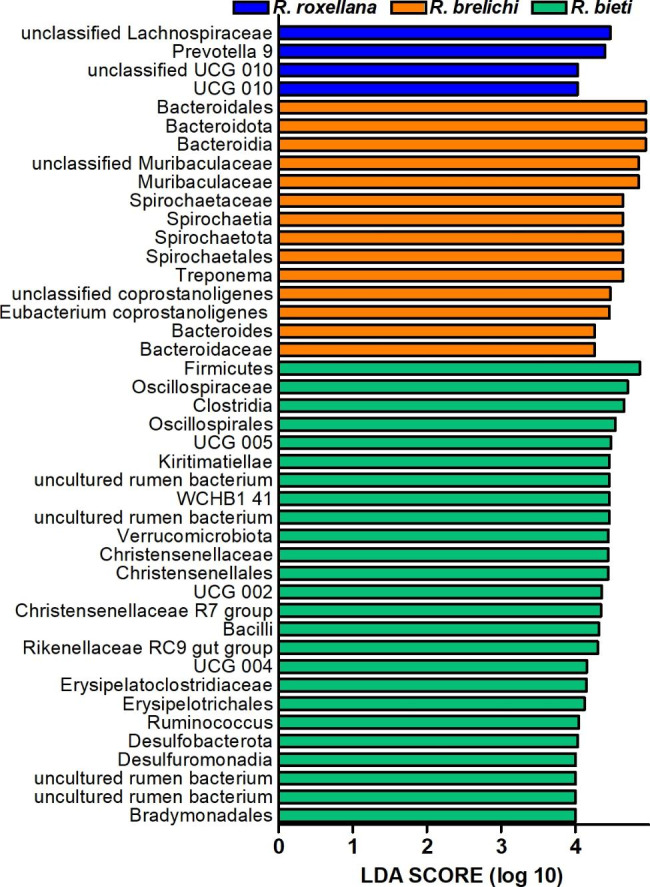
LDA value distribution histogram of the gut microbiota of the three *Rhinopithecus* species. The default LDA threshold is 4.0. Larger LDA values indicate a greater influence of the abundance of bacterial species on the differences in the community among *Rhinopithecus* species

The top ten phyla in the gut microbiota of the three *Rhinopithecus* species were shown in Fig. 4A. The predominant phyla were Firmicutes and Bacteroidetes (87.85% for *R. bieti*, 82.63% for *R. brelichi*, and 79.88% for *R. roxellana*). The relative abundance (average, the same below) of Firmicutes in the gut of *R. bieti* was significantly higher than that of *R. brelichi* (61.75% vs. 47.03%, *P* < 0.01), but it had lower proportion of Bacteroidetes than *R. brelichi* (18.13% vs. 35.60%, *P* < 0.01). Among the nondominant phyla, the proportion of Verrucomicrobiota and Desulfobacterota were higher in *R. bieti* than in *R. brelichi* (Verrucomicrobiota: 7.67% vs. 1.76%, *P* < 0.05; Desulfobacterota: 2.03% vs. 0.13%, *P* < 0.01); the proportion of Spirochaetota was higher in *R. brelichi* than in *R. roxellana* (10.22% vs. 1.30%, *P* < 0.05); the proportion of Elusimicrobiota was higher in *R. roxellana* than in *R. brelichi* (0.33% vs. 0.02%, *P* < 0.01) (Additional file 3). At the genus level, the predominant genera in the gut of *R. bieti*, *R. brelichi* and *R. roxellana* were *UCG 005* (10.38%), *unclassified Muribaculaceae* (16.77%) and *unclassified Lachnospiraceae* (10.09%), respectively (Fig. 4B). Of the top 10 genera in relative abundance, there were five genera with significant differences between *R. bieti* and *R. brelichi* (*unclassified Muribaculaceae*, *UCG 005*, *Eubacterium coprostanoligenes group*, *uncultured rumen bacterium*, and *Ruminococcus*), two genera with significant differences between *R. bieti* and *R. roxellana* (*UCG 002* and *Bacteroides*), and one genus with significant differences between *R. brelichi* and *R. roxellana* (*Treponema*) (Additional file 4). The Linear discriminant analysis effect size (LEfSe) analysis showed that the family and genus-level biomarkers in the gut microbiota of *R. bieti* were *Rikenellaceae RC9 gut group*; *Ruminococcus*; Erysipelatoclostridiaceae and *UCG 004*; Christensenellaceae and *Christensenellaceae R7 group*; Oscillospiraceae, *UCG 002*, and *UCG 005*; and uncultured rumen bacteria from order Bradymonadales and WCHB1-41. Biomarkers enriched in the gut of *R. brelichi* were Bacteroidaceae and *Bacteroides*; Muribaculaceae and *unclassified Muribaculaceae*; Eubacterium coprostanoligenes group and *unclassified Eubacterium coprostanoligenes group*; Spirochaetaceae and *Treponema*. The *Prevotella 9*, *unclassified Lachnospiraceae*, UCG 010 and *unclassified UCG 010* were significantly enriched in the gut of *R. roxellana* (Fig. 5).

### The PICRUSt prediction of functional genes in the gut microbiota

**Fig. 6:**
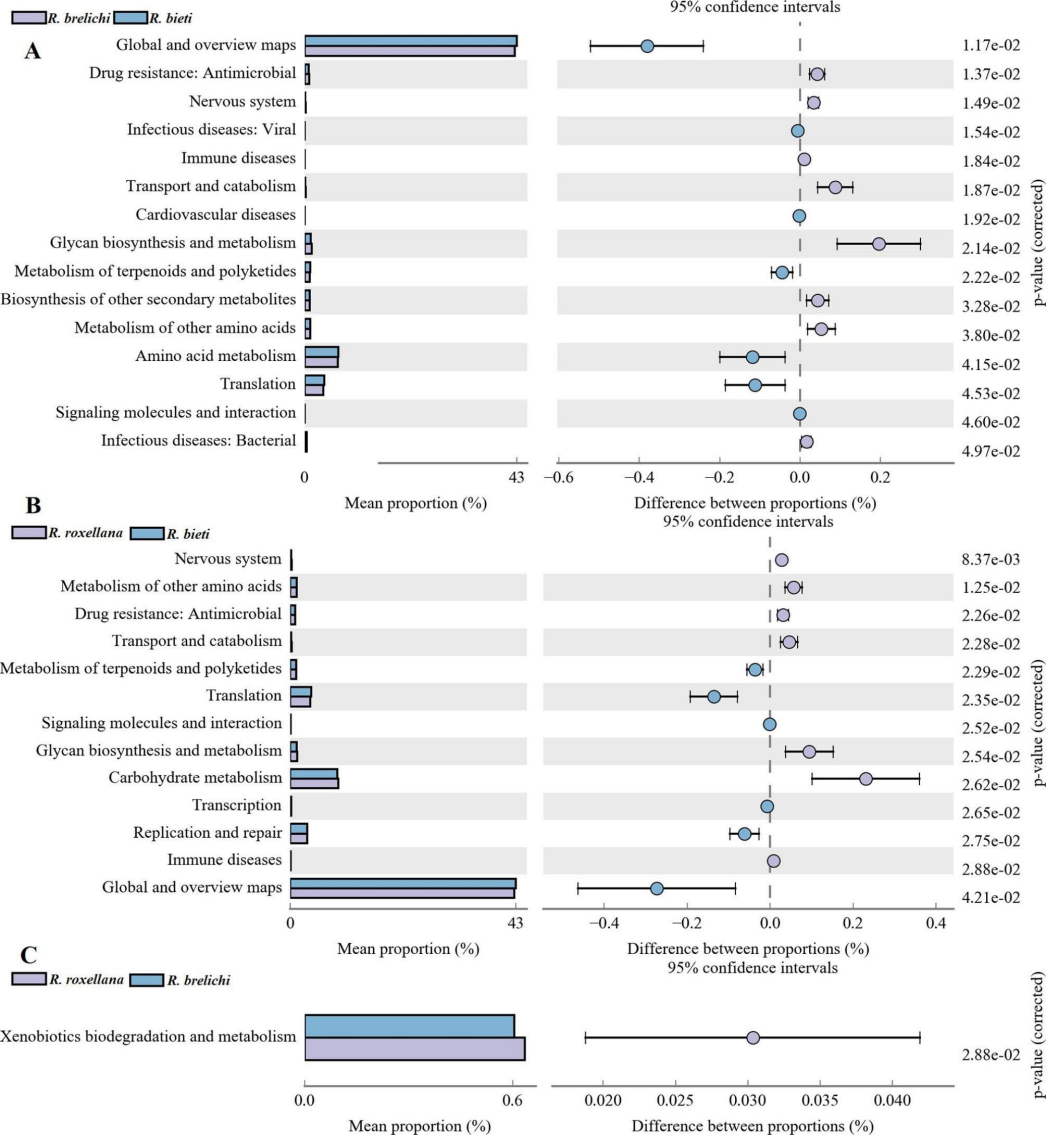
Differences in the KEGG secondary metabolic pathways between three *Rhinopithecus* species. **A** The proportion of different functions between *R. bieti* and *R. brelichi*. **B** The abundance proportion of different functions between *R. bieti* and *R. roxellana*. **C** The abundance proportion of different functions between *R. brelichi* and *R. roxellana*. Software: STAMP. Threshold for significant difference: *P* < 0.05

High-throughput sequencing data for the 16 S rRNA V3-V4 gene were predicted using the Phylogenetic Investigation of Communities by Reconstruction of Unobserved States 2 (PICRUSt 2) metabolic function prediction tool based on the Kyoto Encyclopedia of Genes and Genomes (KEGG). KEGG is a database for understanding high-level functions and utilities of the biological system from molecular-level information. The composition and differential analysis of KEGG metabolic pathways could predict the variations in the functional genes related to the metabolism of the bacterial communities among the three *Rhinopithecus* species. The results showed significant differences in the relative abundance of 15 functional genes between *R. bieti* and *R. brelichi* (Fig. 6A). In the secondary metabolic pathways, amino acid metabolism, metabolism of terpenoids and polyketides, and global and overview maps were significantly higher in *R. bieti* than in *R. brelichi*, while the biosynthesis of other secondary metabolites, the glycan biosynthesis and metabolism, and metabolism of other amino acids were significantly lower than in *R. brelichi*. The relative abundances of 13 functional genes were significantly different between *R. bieti* and *R. roxellana* (Fig. 6B). Similar to *R. brelichi*, the relative abundance of the glycan biosynthesis and metabolism, and metabolism of other amino acids were significantly higher in *R. roxellana* than in *R. bieti*. Additionally, carbohydrate metabolism were also significantly higher in *R. roxellana* than in *R. bieti*. The predicted pathways with significant differences between *R. brelichi* and *R. roxellana* was xenobiotics biodegradation and metabolism. Interestingly, the difference in carbohydrate tertiary metabolism pathways showed that *R. bieti* may have a different carbohydrate metabolism strategies from the other two *Rhinopithecus* species (Additional file 5). In addition to the carbohydrate metabolic pathway, there were no significant differences in other tertiary metabolic pathways between the gut microbiota of *R. brelichi* and *R. roxellana*.

## Discussion

As a nonhuman primate, the study of the microbial community composition and function of snub-nosed monkeys is not only beneficial to protect this endangered species but also to improve the global biodiversity. The present study showed that the predominant phyla of three species of snub-nosed monkeys were Firmicutes and Bacteroidetes, which was consistent with previous studies [22, 23]. Both phyla are closely related to the energy absorption of the host. Most Firmicutes bacteria can use carbohydrates such as cellulose, hemicellulose, and xylan as energy sources [24]. The relative abundance of Firmicutes in *R. bieti* was significantly higher than in *R. brelichi*. Cellulose or hemicellulose-degrading bacterial genera including *Rikenellaceae RC9 gut group* [25], *Ruminococcus* [26], *Christensenellaceae R7 group* [27], *UCG 004* from Erysipelatoclostridiaceae [28], and *UCG 002* and *UCG 005* from Oscillospiraceae [29] were significantly enriched in the gut of *R. bieti*, indicating that *R. bieti* may have a stronger ability to use plant fibers as an energy source than the other two snub-nosed monkeys. The predicted microbiota gene function was consistent with the characteristics of the bacterial communities composition of the above: the proportion of the propanoate and butanoate metabolism pathway was significantly higher in *R. bieti*. The genomes of Bacteroidetes contain rich polysaccharide lyase and glycoside hydrolyase genes, which is why it is considered to be the main degraders of polysaccharides [30, 31]. The Bacteroidetes had the highest abundance in the gut of *R. brelichi*. Muribaculaceae and Bacteroidaceae were enriched in *R. brelichi* and *Prevotella 9* was enriched in *R. roxellana.* Muribaculaceae bacteria are the main users of mucosal sugar [32]. Members of Bacteroidaceae provide nutrients to the host by breaking down different glycans [33, 34]. In the secondary metabolic pathways, the proportion of the glycan biosynthesis and metabolism pathway in *R. brelichi* and *R. roxellana* was significantly higher than in *R. bieti*. In particular, other important carbohydrate tertiary metabolism pathways such as amino sugar and nucleotide sugar metabolism, ascorbate and aldarate metabolism, galactose metabolism, pentose and glucuronate interconversions, and starch and sucrose metabolism were significantly enriched in *R. brelichi* or *R. roxellana*. These results suggest that different species of snub-nosed monkeys may have different strategies for carbohydrate metabolism and future studies employing a larger sample size, more species of snub-nosed monkeys, and omics sequencing approaches can evaluate this hypothesis.

In the nondominant phyla, Verrucomicrobiota was significantly enriched in *R. bieti*. Both Kiritimatiellae, WCHB1-41, and *uncultured rumen bacterium* under phylum Verrucomicrobiota were significantly enriched in *R. bieti*. The arginine and fatty acid biosynthesis pathways encoded by the WCHB 1–41 genome are involved in host energy metabolism and nitrogen utilization in response to nutrient deficiencies caused by high altitude and harsh cold [35]. Comparison of lipid metabolism pathways among the three species of snub-nosed monkeys showed that the proportion of fatty acid metabolism pathway in the gut microbiota of *R. bieti* was significantly higher than that of *R. brelichi* and *R. roxellana* (*R. bieti* 0.63% vs. *R. brelichi* 0.58%, *P* < 0.05; *R. bieti* 0.63% vs. *R. roxellana* 0.58%, *P* < 0.05). The phylum Spirochaetota was significantly enriched in *R. brelichi*. *Treponema* was the main genus responsible for this difference (10.22% for *R. brelichi* vs. 1.30% for *R. roxellana, P* < 0.05). *Treponema* has enzymes that mediate pyruvate oxidation and decarboxylation to enter the citrate cycle, promoting the biosynthesis of arginine and fatty acids [36]. It has been suggested that low-abundance bacteria that are not normally part of the core community are drivers of changes in the composition of the host post-gut microbiota [37]. These differential low abundance bacterial communities in this study not only caused differences in the composition of the consortium, but also may play a relevant role in interspecific difference in metabolic pathways.

Long-term habitat and dietary differences encourage differentiated hosts to adopt different strategies to acquire microbes from nature, eventually reaching a healthy and balanced symbiotic relationship [38]. This symbiotic relationship makes species-specific microbial communities somewhat resistant to disturbance [39]. An analysis of adaptive variations in the gut microbiota of 18 nonhuman primates revealed that the physiological evolution of the host has a stronger effect in the construction of the microbiota than the dietary niche [40]. All snub-nosed monkeys in the present study were kept at Beijing Zoo with the same living environment and dietary structure, which minimized the influence of environmental and dietary factors on the gut microbiota. All samples shared 24.70% of the ASVs, mainly annotated to Firmicutes and Bacteroidetes, indicating that snub-nosed monkeys have the same predominant phyla. PCoA and UPGMA clustering analysis showed that *R. brelichi* and *R. roxellana* had a more similar microbial community composition. The *R*^2^ value of the PERMANOVA analysis and *R* values of the ANOSIM analysis were both minimal in the comparisons of beta diversity between *R. brelichi* and *R. roxellana*, indicating that the similarity of gut microbiota was highest between these two species. The differential analysis of the KEGG metabolic pathways among *Rhinopithecus* species also did not show significant differences in the metabolic function of these two species. The gut microbiota of *R. bieti* showed greater differences in both composition and predicted functions. Therefore, the profound effects of host species on gut microbiota stability and adaptability must be considered when studying nutritional strategies or the effects of niche on the gut microbiota in snub-nosed monkeys.

The present study has two limitations: (1) The small sample size (both in terms of the number of host species represented and the number of samples) could not meet the representativeness of the gut microbiota characteristics of the host species, so subsequent studies need to increase the sample size and host species to enhance its representativeness. (2) The PICRUSt 2 software used to predict metagenomics function based on 16 S genes has limitations in identifying rare environment-specific functions and distinguishing strain-specific functionality. In the future, more precise information on the microbial composition and genes involved in metabolism can be obtained through shotgun metagenomics to explore the mechanisms of interaction between gut microbiota and host metabolism.

## Conclusions

This study investigated the characteristics of the gut microbiota of endangered *R. bieti*, *R. brelichi*, and *R. roxellana* under the same captive conditions. The predominant phyla of the three *Rhinopithecus* species were Firmicutes and Bacteroidetes, but the proportion and the species composition under the phylum were different. As most of the Firmicutes and Bacteroidetes species participated in carbohydrate metabolism, there were significant differences in carbohydrate metabolism pathways between *R. bieti* and the other two snub-nosed monkeys. The *R. brelichi* and *R. roxellana*, which belong to the Northern species, also have a higher similarity in their gut microbiota composition and predicted metabolic functions. The gut microbiota of *R. bieti* belonging to the Himalayan species showed different composition and predicted functions. In conclusion, we obtained the gut microbiota characteristics of endemic and endangered snub-nosed monkeys in China under captive conditions. Through real-time monitoring of microbiota changes, it can provide data for disease monitoring and artificial feed research, to achieve the purpose of conserving endangered snub-nosed monkeys.

## Materials and methods

### Sample collection

Fresh feces from *R. bieti* (males, 3–9 years old, N = 5), *R. brelichi* (males, 2–8 years old, N = 5) and *R. roxellana* monkeys (males, 5–7 years old, N = 5) were collected from Beijing Zoo in January 2022. The three *Rhinopithecus* species were captived in adjacent enclosures and provided with the same food. The food composition of these monkeys includes fresh food (leaves, fruits, vegetables, etc.) and cooked food (corn cakes, eggs, and beef strips, etc.). After defecation, the middle part of the monkey feces was clamped in sterile eppendorf tubes, then transported to the laboratory.

### Total DNA extraction and NovaSeq sequencing of the fecal microbiota

The total DNA from the fecal microbiota was extracted using the GenElute™ Stool DNA Isolation Kit (Sigma-Aldrich, USA) according to the instructions. DNA quality was detected using a Nanodrop 2000 ultra-trace spectrophotometer (Thermo Scientific, USA) and 1% agarose gel electrophoresis. The 16 S rRNA V3-V4 was amplified using universal primers (338 F and 806R) [41], three repeats for each sample, then triplicate products were mixed, electrophoresed in 2% agarose gel at 110 V for 20 min for quality detection. The PCR recovered products were mixed equally according to their concentration. High-throughput sequencing (250 bp, paired-end) was performed using the Illumina NovaSeq platform after clone libraries were constructed using the TruSeq® Nano DNA Kit (Illumina, USA).

### Bioinformatics analysis

Adapters and low-quality sequences with read length less than 50 bp were removed using Trimmomatic v0.33 software [42]. Primer sequences were identified and removed following the parameters with 20% of maximum mismatch and 80% of minimum coverage using the Cutadapt v1.9.1 software [43]. The quality control data was denoised using DADA2 v1.20 workflow [44] in QIIME 2 v2020.6 software [45]. The filtering threshold for ASVs was set to 0.005% of the number of all sequences. Species annotation was performed using the “classify-sklearn” function based on the Naive Bayesian classifier in the QIIME 2 software, with 0.7 set as the confidence threshold. The Silva database (Release138, https://www.arb-silva.de/) [46] was searched to obtain the taxonomic information of the ASV representative sequences. The values of the alpha diversity index (Abundance based coverage estimator- ACE, Shannon diversity) for the samples were calculated using the QIIME 2 v2020.6 software. Differences in alpha diversity and composition of gut microbiota between the three *Rhinopithecus* species were analyzed using Kruskal Wallis rank-sum test, and *P*-values were corrected using the Benjamini-Hochberg method in the vegan package of R software v3.6.1. The PCoA [47] and UPGMA [48] clustering tree based on the weighted UniFrac distances (comparisons based on the phylogenetic tree) were used to assess the beta diversity between the three *Rhinopithecus* species. The significance of microbiota differences and similarities between the three *Rhinopithecus* species were verified using PERMANOVA analysis [49] and ANOSIM analysis [50] based on the weighted UniFrac distances in the vegan package of R software v3.6.1. LEfSe [51] analysis was used to search for biomarkers that would distinguish each host species. Clustering ASVs information was compared with the sequenced microbial genome database using PICRUSt 2 software [52] to obtain the functional types and abundance of the corresponding species in the KEGG database (https://www.kegg.jp/) [53]. Differences in KEGG pathways between the three *Rhinopithecus* species were analyzed using Statistical Analysis of Metagenomic Profiles (STAMP) v2.1.3 [54], and the false discovery rate was controlled by Benjamini-Hochberg procedure.

## Electronic supplementary material

Below is the link to the electronic supplementary material.

Supplementary Material 1

Supplementary Material 2

Supplementary Material 3

Supplementary Material 4

Supplementary Material 5

## Data Availability

The datasets presented in this study can be found in online repositories. The names of the repository and accession number(s) can be found below: Sequence Read Archive (NCBI, USA), PRJNA934657.
