# Supplementary material for: Comparative study of the gut microbiota in three captive Rhinopithecus species
Source: BMC Genomics. 2023 Jul 14;24:398. doi: 10.1186/s12864-023-09440-z (PMC10349479; doi:10.1186/s12864-023-09440-z)
Supplement: Supplementary file 1 — Supplementary Material 1 [file 12864_2023_9440_MOESM1_ESM.docx]

Supplementary Table S1 Raw data processing results

| Sample  ID | Raw  Reads | Clean  Reads | Denoised Reads | Merged Reads | Non-chimeric Reads |
| --- | --- | --- | --- | --- | --- |
| *R. bieti* 1 | 79,871 | 79,655 | 78,052 | 75,401 | 73,274 |
| *R. bieti* 2 | 80,425 | 80,200 | 77,934 | 69,182 | 64,972 |
| *R. bieti* 3 | 79,798 | 79,569 | 78,101 | 73,664 | 71,250 |
| *R. bieti* 4 | 80,247 | 80,020 | 77,969 | 72,154 | 69,732 |
| *R. bieti* 5 | 80,307 | 80,059 | 78,381 | 73,481 | 71,146 |
| *R. brelichi* 1 | 80,062 | 79,835 | 78,039 | 72,304 | 69,084 |
| *R. brelichi* 2 | 82,979 | 82,712 | 80,011 | 70,472 | 62,291 |
| *R. brelichi* 3 | 80,134 | 79,902 | 78,822 | 76,316 | 74,359 |
| *R. brelichi* 4 | 79,853 | 79,637 | 78,129 | 74,338 | 72,401 |
| *R. brelichi* 5 | 80,044 | 79,825 | 78,059 | 73,657 | 69,899 |
| *R. roxellana* 1 | 80,383 | 80,151 | 78,703 | 74,175 | 72,067 |
| *R. roxellana* 2 | 79,925 | 79,703 | 78,200 | 73,596 | 71,541 |
| *R. roxellana* 3 | 79,884 | 79,669 | 77,532 | 69,919 | 66,699 |
| *R. roxellana* 4 | 79,980 | 79,786 | 77,954 | 71,044 | 68,228 |
| *R. roxellana* 5 | 80,198 | 79,979 | 78,218 | 72,370 | 70,062 |
