# Supplementary figures and images for: Comparative study of the gut microbiota in three captive Rhinopithecus species
Source: BMC Genomics. 2023 Jul 14;24:398. doi: 10.1186/s12864-023-09440-z (PMC10349479; doi:10.1186/s12864-023-09440-z)

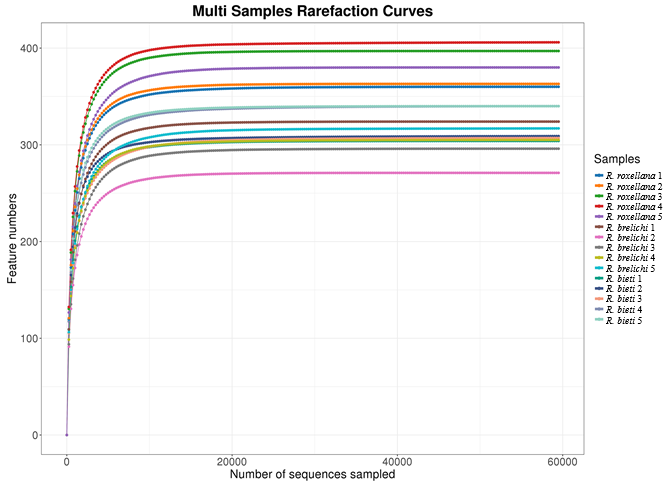


**Supplementary Fig. S1** Rarefaction curves of gut microbiota of the three *Rhinopithecus* species.

Supplement: Supplementary file 2 — Supplementary Material 2 [file 12864_2023_9440_MOESM2_ESM.docx]
