# Supplementary material for: Comparative study of the gut microbiota in three captive Rhinopithecus species
Source: BMC Genomics. 2023 Jul 14;24:398. doi: 10.1186/s12864-023-09440-z (PMC10349479; doi:10.1186/s12864-023-09440-z)
Supplement: Supplementary file 3 — Supplementary Material 3 [file 12864_2023_9440_MOESM3_ESM.docx]

Supplementary Table S2 Differences in relative abundance of the top 10 phyla between the three *Rhinopithecus* species (Kruskal Wallis rank-sum test, and *P*-values were corrected using the Benjamini-Hochberg method. ns: *P* > 0.05, no significance).

| Phylum | *R. bieti*  (%) | *R. brelichi*  (%) | *R. roxellana*  (%) | *R. bieti* vs  *R. brelichi* (*P*) | *R. bieti* vs  *R. roxellana* (*P*) | *R. brelichi* vs  *R. roxellana* (*P*) |
| --- | --- | --- | --- | --- | --- | --- |
| Firmicutes | 61.75 | 47.03 | 57.04 | *P*<0.01 | ns | ns |
| Bacteroidota | 18.13 | 35.60 | 30.82 | *P*<0.01 | ns | ns |
| Spirochaetota | 6.23 | 10.22 | 1.30 | ns | ns | *P*<0.05 |
| Verrucomicrobiota | 7.67 | 1.76 | 2.57 | *P*<0.05 | ns | ns |
| Proteobacteria | 1.41 | 1.44 | 2.77 | ns | ns | ns |
| unclassified Bacteria | 1.50 | 2.54 | 1.03 | ns | ns | ns |
| Cyanobacteria | 0.42 | 0.61 | 1.86 | ns | ns | ns |
| Fibrobacterota | 0.50 | 0.51 | 1.70 | ns | ns | ns |
| Desulfobacterota | 2.03 | 0.13 | 0.37 | *P*<0.01 | ns | ns |
| Elusimicrobiota | 0.11 | 0.02 | 0.33 | ns | ns | *P*<0.01 |
