# Supplementary material for: Comparative study of the gut microbiota in three captive Rhinopithecus species
Source: BMC Genomics. 2023 Jul 14;24:398. doi: 10.1186/s12864-023-09440-z (PMC10349479; doi:10.1186/s12864-023-09440-z)
Supplement: Supplementary file 4 — Supplementary Material 4 [file 12864_2023_9440_MOESM4_ESM.docx]

Supplementary Table S3 Differences in relative abundance of the top 10 genera between the three *Rhinopithecus* species (Kruskal Wallis rank-sum test, and *P*-values were corrected using the Benjamini-Hochberg method. ns: *P* > 0.05, no significance).

| Genus | *R. bieti*  (%) | *R. brelichi*  (%) | *R. roxellana*  (%) | *R. bieti* vs  *R. brelichi* (*P*) | *R. bieti* vs  *R. roxellana* (*P*) | *R. brelichi* vs  *R. roxellana* (*P*) |
| --- | --- | --- | --- | --- | --- | --- |
| *unclassified Muribaculaceae* | 2.58 | 16.77 | 8.55 | *P*<0.01 | ns | ns |
| *UCG 005* | 10.38 | 4.66 | 6.56 | *P*<0.05 | ns | ns |
| *unclassified Lachnospiraceae* | 4.31 | 4.34 | 10.09 | ns | ns | ns |
| *Treponema* | 6.23 | 10.22 | 1.30 | ns | ns | *P*<0.05 |
| *Eubacterium coprostanoligenes group* | 2.29 | 8.11 | 6.40 | *P*<0.01 | ns | ns |
| *uncultured rumen bacterium* | 10.02 | 1.86 | 4.78 | *P*<0.05 | ns | ns |
| *Christensenellaceae R7 group* | 6.49 | 5.76 | 2.30 | ns | ns | ns |
| *UCG 002* | 6.45 | 3.18 | 2.03 | ns | *P*<0.05 | ns |
| *Bacteroides* | 1.48 | 4.84 | 4.82 | ns | *P*<0.05 | ns |
| *Ruminococcus* | 4.03 | 1.68 | 2.61 | *P*<0.05 | ns | ns |
