# Supplementary material for: Comparative study of the gut microbiota in three captive Rhinopithecus species
Source: BMC Genomics. 2023 Jul 14;24:398. doi: 10.1186/s12864-023-09440-z (PMC10349479; doi:10.1186/s12864-023-09440-z)
Supplement: Supplementary file 5 — Supplementary Material 5 [file 12864_2023_9440_MOESM5_ESM.docx]

Supplementary Table S4 Differences in Carbohydrate tertiary metabolic pathways between the gut microbiota of the three *Rhinopithecus* species (Kruskal Wallis rank-sum test, and *P*-values were corrected using the Benjamini-Hochberg method. ns: *P* > 0.05, no significance).

| Metabolic pathway | *R. bieti*  (%) | *R. brelichi*  (%) | *R. roxellana*  (%) | *R. bieti* vs  *R. brelichi* (*P*) | *R. bieti* vs  *R. roxellana* (*P*) | *R. brelichi* vs  *R. roxellana* (*P*) |
| --- | --- | --- | --- | --- | --- | --- |
| Amino sugar and nucleotide sugar metabolism | 1.07 | 1.10 | 1.11 | ns | *P*<0.05 | ns |
| Ascorbate and aldarate metabolism | 0.05 | 0.06 | 0.06 | ns | *P*<0.05 | ns |
| Butanoate metabolism | 0.61 | 0.57 | 0.57 | *P*<0.05 | *P*<0.05 | ns |
| C5-Branched dibasic acid metabolism | 0.27 | 0.26 | 0.26 | ns | *P*<0.05 | ns |
| Citrate cycle (TCA cycle) | 0.62 | 0.60 | 0.59 | ns | ns | ns |
| Fructose and mannose metabolism | 0.60 | 0.63 | 0.64 | ns | ns | ns |
| Galactose metabolism | 0.49 | 0.62 | 0.61 | *P*<0.05 | *P*<0.05 | ns |
| Glycolysis / Gluconeogenesis | 1.04 | 1.04 | 1.04 | ns | ns | ns |
| Glyoxylate and dicarboxylate metabolism | 0.66 | 0.69 | 0.68 | ns | *P*<0.05 | ns |
| Inositol phosphate metabolism | 0.12 | 0.12 | 0.12 | ns | ns | ns |
| Pentose and glucuronate interconversions | 0.28 | 0.31 | 0.32 | ns | *P*<0.01 | ns |
| Pentose phosphate pathway | 0.72 | 0.73 | 0.75 | ns | ns | ns |
| Propanoate metabolism | 0.58 | 0.53 | 0.54 | *P*<0.05 | *P*<0.05 | ns |
| Pyruvate metabolism | 1.10 | 1.08 | 1.08 | ns | ns | ns |
| Starch and sucrose metabolism | 0.74 | 0.80 | 0.82 | ns | *P*<0.05 | ns |
